# Determining Existing Human Population Immunity as Part of Assessing Influenza Pandemic Risk

**DOI:** 10.3201/eid2805.211965

**Published:** 2022-05

**Authors:** Jonathan Tin Lai Cheung, Tim K. Tsang, Hui-ling Yen, Ranawaka A.P.M. Perera, Chris Ka Pun Mok, Yong Ping Lin, Benjamin J. Cowling, Malik Peiris

**Affiliations:** University of Hong Kong, Hong Kong (J.T.L. Cheung, T.K. Tsang, H. Yen, R.A.P.M. Perera, C.K.P. Mok, B.J. Cowling, M. Peiris);; Chinese University of Hong Kong, Hong Kong (C.K.P. Mok);; First Affiliated Hospital of Guangzhou Medical University, Guangdong, China (Y.P. Lin)

**Keywords:** influenza, swine flu, pandemics, risk assessment, seroprevalence, hemagglutinin, neuraminidase, hemagglutination inhibiting antibody, swine, population immunity, reproduction numbers, viruses, zoonoses, China, Hong Kong

## Abstract

Zoonotic influenza infections continue to threaten human health. Ongoing surveillance and risk assessment of animal viruses are needed for pandemic preparedness, and population immunity is an important component of risk assessment. We determined age-stratified hemagglutinin inhibition seroprevalence against 5 swine influenza viruses circulating in Hong Kong and Guangzhou in China. Using hemagglutinin inhibition seroprevalence and titers, we modeled the effect of population immunity on the basic reproduction number (R_0_) if each virus were to become transmissible among humans. Among 353 individual serum samples, we reported low seroprevalence for triple-reassortant H1N2 and Eurasian avian-like H1N1 influenza viruses, which would reduce R_0_ by only 18%–20%. The smallest R_0_ needed to cause a pandemic was 1.22–1.24, meaning existing population immunity would be insufficient to block the spread of these H1N1 or H1N2 variants. For human-origin H3N2, existing population immunity could suppress R_0_ by 47%, thus reducing pandemic risk.

An influenza pandemic can occur when an influenza A virus with gene segments derived in part or whole from animal viruses becomes able to efficiently and sustainably transmit among humans (*1*,*2*). Lack of prior immunity among the human population to the hemagglutinin (HA) of a novel virus enables pandemic spread of that virus. New influenza vaccines require >7 months to develop, but pandemics spread faster than that; a new vaccine would not be available in time to prevent a first pandemic wave, as was seen during the 2009 influenza (H1N1) pandemic (*1*,*3*). Because of this delay, surveillance and risk assessment are used to anticipate pandemic threats (*4*,*5*), enabling preemptive vaccine development to be initiated. Prepandemic actions might include developing vaccine seed strains, experimental vaccine seed lots, or even phase 1 clinical trials of prepandemic vaccine candidates, depending on risk assessment data. The World Health Organization (WHO) and Centers for Disease Control and Prevention (CDC) developed the Tool for Influenza Pandemic Risk Assessment and Influenza Risk Assessment Tool in response to the need for standardized and transparent tools to assess the pandemic potential of influenza viruses (*5*,*6*). Based on the properties of the virus, attributes in the human population, and virus ecology in animal hosts (*6*), such assessments attempt to determine emergence risk, the potential of an animal virus to become able to efficiently transmit among humans, and effect risk, the effect and severity if that virus were to spread among humans. Population immunity is an important feature of assessing risk.

Pandemic spread depends on the ability of a virus to transmit among humans, which is measured as the basic reproduction number (R_0_), the average number of secondary cases generated by 1 infected person in a completely susceptible population. If R_0_ is ≥1, the outbreak will tend to spread or persist, but if R_0_ is <1, the outbreak will likely not spread or persist. At the start of some pandemics, such as the H1N1 pandemic in 2009, immunity levels may differ among some age groups, and the effective reproduction number, R_t_, better reflects transmissibility. This value depends on virus characteristics (biological transmissibility), population density and social mixing, and existing human population immunity, which can reduce transmission efficiency. Existing cross-reactive population immunity is a key factor that can inhibit the spread of the virus among humans and also one key risk element for assessing emergence risk.

Hemagglutination inhibition (HAI) antibody is a well-established immune correlate of protection against influenza. Data from experimentally infected humans show a correlation between increasing HAI titer to an influenza A virus and decreasing probability of infection; ≈50% of persons protected at an HAI titer of 40 became infected (*7*,*8*). However, there is a gradient of protection above and below this threshold HAI titer of 40. Estimates of population immunity in risk assessment algorithms would benefit from greater precision and scientific rationale (*6*). Current algorithms do not use the range or age-stratified distribution of HAI titers in the population, which might affect measures of overall population immunity. In a previous study (*9*), we assessed the effect on the R_t_ of age-stratified distribution of HAI titers to H2N2 influenza viruses. In this study, we refined and extended this approach, including the use of data on antibody titers, and applied it to assess human population immunity to swine influenza viruses (SIVs).

Eurasian avian (EA)–like H1 SIVs have circulated in China since 2001 (*10*) and have been the dominant strain in southern China since 2005 (*11*). Triple-reassortant internal gene (TRIG) H1 SIVs from North America have been detected in swine in China since 2002 and Vietnam since 2011 (*12*). Swine carry pandemic H1N1 virus gene segments acquired by reassortment (*11*,*13*–*15*).

China and Vietnam are the largest swine producers in Asia and together account for 40.2% of global production (https://www.statista.com/statistics/273232/net-pork-production-worldwide-by-country). Swine are often raised in close proximity to avian species and humans, with low biosecurity, enhancing risks of pandemic emergence (*1*,*4*). In this study, we assessed age-stratified levels of HAI antibodies to swine influenza A viruses recently circulating in China in human serum samples collected in Hong Kong and Guangzhou, then used these data to quantify population immunity to infection. In addition, as a case study, we modeled pre-2009 population immunity to the 2009 H1N1 virus (H1N1pdm09) as an example of an actual swine virus that emerged in pandemic form (*16*).

## Methods

### Cross-Sectional Age-Stratified Serum Panels

We used serum samples collected December 6, 2013–March 29, 2014 from children and adults in Hong Kong as part of a community-based cohort study (*17*). We recruited study participants on the household level, identifying households using random digit dialing. The study protocol was approved by the institutional review board of the University of Hong Kong.

We selected an age-stratified subset of 173 serum samples from this larger study for the present investigation. We selected an additional age-stratified panel of 180 anonymized serum samples from residual serum samples from patients with nonrespiratory and noninfectious illnesses admitted to the First Affiliated Hospital of Guangzhou Medical University, February 9–March 31, 2015. The study was approved by the ethics committee of the First Affiliated Hospital of Guangzhou Medical University (reference no. 2015-8). 

### Virus Antigens

As antigens for HAI tests, we selected 5 H1 and H3 subtype swine influenza viruses representing predominant lineages of viruses circulating in China: EA H1 swine virus A/swine/Hong Kong/NS4003/2016 (H1N1)(NS4003); TRIG H1-lineage virus A/swine/Hong Kong/NS301/2013 (H1N2)(NS301); H1N1pdm09-like swine H1N1 virus A/swine/Hong Kong/1436/2016 (H1N1) (TS1436); and a Binh Duong-like H3N2 swine virus A/swine/Hong Kong/4348/2016 (H3N2) (TS4348), which originated from the human H3N2 seasonal viruses in 2004–2006 (Appendix Figure 1) (13,*18*). The fifth lineage was a recombinant virus we generated, EA-lineage A/swine/Guangdong/104/2013 (H1N1) (GD104), reported elsewhere to have low cross-reactivity with human serum samples (*19*). We synthesized the HA gene of wild-type GD104 virus (GenBank accession no. KJ725040), cloning it into the pHW2000 vector (*20*,*21*) and a recombinant virus A/PR/8/34^PB2,PB1,PA,NP,NA,M,NS^ × A/swine/Guangdong/104/2013^HA^ (Rg-PR8 × GD104^HA^) containing the HA gene derived from A/swine/Guangdong/104/2013 (H1N1) (GD104) and the 7 other genes from A/PR/8/34 (H1N1), rescued by virus reverse genetics (Appendix) (*21*). We also recorded the origins of the 8 gene segments of each virus (Appendix Figure 2). We propagated the SIVs in MDCK cells as described elsewhere (*14*).

### HAI Assay

We pretreated serum samples with receptor-destroying enzyme (Denka Seiken, https://www.denka.co.jp), followed by heat inactivation at 56°C for 30 min, then serially diluted treated serum samples 2-fold (1:10–1:1,280) into microtiter plates. We performed HAI with 0.5% turkey red blood cells using an equal volume of virus with 8 HA units/50 μL in duplicate (*22*). We determined HAI titer by the highest dilution of serum that prevented complete hemagglutination.

For calculating geometric mean titers (GMTs), we assigned a value of 5 to serum samples with a titer <10 and a value of 1,280 to those with a titer ≥1,280. We used antibody titers of 10 and 40 as cutoff values and used the Fisher exact test to compare the differences in seroprevalence between groups. We considered differences with a p value <0.05 statistically significant. We conducted all statistical analyses using R version 3.6.1 (https://cran.r-project.org/bin/windows/base/old/3.6.1). 

### Reproduction Number Modeling

We partitioned the seroprevalence data into 8 age groups by decade (e.g., 0–10 y, 11–20 y) and 9 HAI titer levels: <10, 10, 20, 40, 80, 160, 320, 640, and ≥1,280. We obtained population age distribution from the most recent census data from Hong Kong (2016; https://www.censtatd.gov.hk/en/scode459.html) and Guangzhou (2015; http://tjj.gz.gov.cn/pchb/2015n1rkcydc/content/post_2787426.html). We used data from a human challenge study to determine the protection against infection associated with each HAI antibody titer (*7*,*23*), then estimated the proportion of population in each HAI titer group for each age group using Bayesian inference with Dirichlet conjugates for multinomial likelihood assuming noninformative priors (Appendix). We calculated the proportion of the population that was immune by weighting the age-stratified sample immunity profile to the corresponding population age structure. We then constructed the next-generation transmission matrix using the social contact matrix for Hong Kong (*24*) and used the social contact matrix for the UK population for comparison (*25*). We defined R_0_ as the largest eigenvalue of the transmission matrix (*26*,*27*), then constructed another transmission matrix in which we subtracted the population protected by HAI antibodies from the total, thus including only the susceptible population from each age group, meaning R_t_ was the largest eigenvalue of this matrix. Given that population immunity profile, we calculated the corresponding relative reduction in transmissibility, then computed the smallest R_0_ needed to cause a pandemic for each test virus. We generated 95% credible intervals (CrI) for the estimated parameters using 10,000 repeated samples randomly drawn from the joint posterior distributionfor each age group (Appendix).

### Historical Pandemic Strain Simulation

To test our methodology on data from an actual recent pandemic, we used the same methods to assess population immunity to H1N1pdm09 in human serum samples collected before its spread in Hong Kong. Prior to the emergence of the 2009 pandemic, only those >50 years of age had cross-reactive HAI antibodies to H1N1pdm09 at a seroprevalence of >10% (*16*,*28*). We retrieved A/California/4/2009 HAI data from 2 serologic surveys performed in the population of Hong Kong in November–December 2008 and July–August 2009, before the onset of the first wave of the 2009 pandemic in Hong Kong (*29*,*30*). We imputed those HAI data into our reproduction number model to assess all-age population serologic immunity and susceptibility in a prepandemic setting against a virus of proven pandemic potential. We also retrieved HAI data on the H2N2 pandemic strain A/Singapore/1/57(H2N2) from a serologic survey conducted in Hong Kong in 2011 (*9*). Only those persons born before 1968 would be expected to carry detectable antibodies for the H2N2 viruses. We used methods from this study to assess the effect of current age-specific human population immunity against a H2-subtype influenza virus if it were to reemerge as a pandemic strain.

## Results

### Age-Stratified Seroprevalence

**Figure 1:**
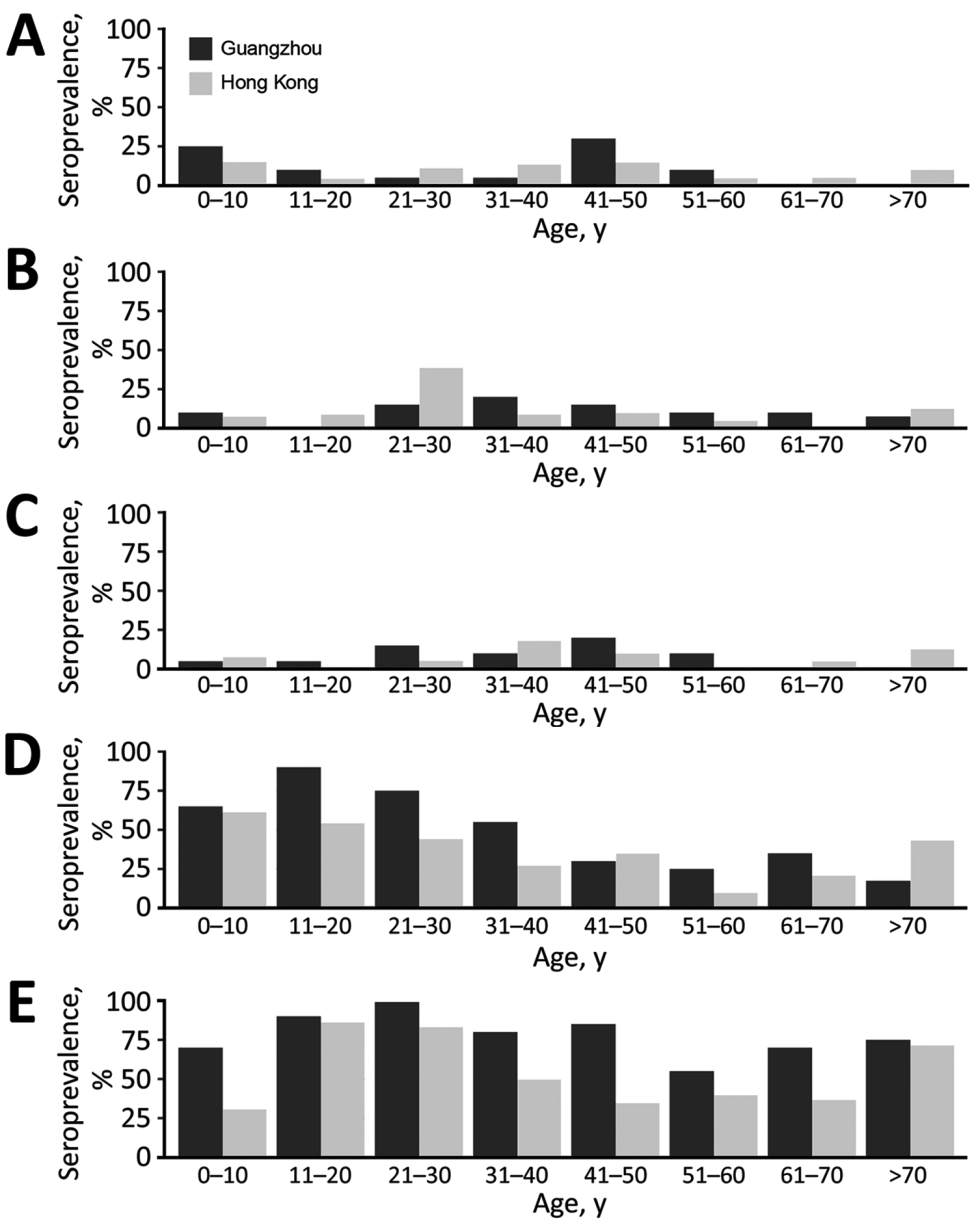
Seroprevalence of hemagglutination inhibition antibodies to different swine influenza viruses, by age group and location, in study to determine existing human population immunity as part of assessing influenza pandemic risk. A) A/swine/Hong Kong/NS4003/2016 (EA); B) A/swine/Guangdong/104/2013 (EA); C) A/swine/Hong Kong/NS301/2013 (TR); D) A/swine/Hong Kong/1436/2016 (pdm09); E) A/swine/Hong Kong/4348/2016 (BD-like H3). BD, Binh Duong; EA, Eurasian avian-like; pdm09, 2009 pandemic strain; TR, triple-reassortant.

Among serum samples with HAI titers ≥40 from the Hong Kong and Guangzhou (Figure 1), stratified by 10-year age intervals, we found no significant differences across all age groups in the seroprevalence to A/Sw/HK/NS4003/2016 (H1N1), A/Sw/GD/104/2013 (H1N1), A/Sw/HK/NS301/2013 (H1N2), or A/Sw/HK/1436/2016 (H1N1). We found a significant difference in the seroprevalence of A/Sw/HK/4348/2016 (H3N2) virus HAI only in the age group 41–50 years; seroprevalence was significantly higher in serum samples from Guangzhou than Hong Kong (p = 0.003). Considering the overall similarity of the patterns of seroprevalence in Hong Kong and Guangzhou, we combined data from the 2 cities for further analysis to assess population-level immunity.

Data on the overall HAI seroprevalence at titers of ≥10 and ≥40 and GMTs of antibodies to 5 tested viruses overall (Table 1 and age-stratified data (Table 2 showed an overall low seroprevalence to 2 H1N1 EA viruses and the H1N2 TRIG virus. In contrast, 41.4% of samples had antibody titers ≥40 to H1N1pdm09-like virus (Table 1); we found greater seroprevalence levels in children and younger adults <30 years of age (Table 2). Overall, >67% of persons from Hong Kong and Guangzhou had titers >40 to the Binh Duong-like H3N2 virus A/Sw/HK/4348/2016, the predominant H3N2 virus lineage circulating in China and Vietnam, which has an HA derived from seasonal influenza viruses that circulated in humans in 2004. Persons in age groups 11–20 and 21–30 years had higher seroprevalence and GMT (Table 2).

**Table 1:** Seroprevalence and geometric mean titer for swine influenza viruses of H1 and H3 subtype in serum specimens from 353 persons in Hong Kong and Guangzhou, China*

Virus	Virus abbreviation	Virus lineage	No. (%) persons		GMT
			Seroprevalence ≥40	Seroprevalence ≥10	
A/swine/HK/NS4003/2016 (H1N1)	NS4003	EA	34 (9.6)	105 (29.7)	7.67
A/swine/GD/104/2013 (H1N1)	GD104	EA	39 (11.0)	89 (25.2)	7.84
A/swine/HK/NS301/2013 (H1N2)	NS301	TRIG	27 (7.6)	115 (32.6)	7.76
A/swine/HK/1436/2016 (H1N1)	TS1436	Pandemic (pdm09)	146 (41.4)	222 (62.9)	20.96
A/swine/HK/4348/2016 (H3N2)	TS4348	Seasonal (BD-like H3)	239 (67.7)	308 (87.3)	48.77

*Serum samples were collected during 2013–2014 in Hong Kong and during 2015 in Guangzhou. BD, Binh Duong; EA, Eurasian avian-like; GMT, geometric mean titer; TRIG, triple-reassortant internal gene.

**Table 2:** Age-stratified seroprevalence and GMT to swine influenza viruses of different lineages among 353 persons in Hong Kong and Guangzhou, China*

Patient age, y	NS4003 EA, H1N1			GD104 EA, H1N1			NS301 TRIG, H1N2			TS1436 H1N1pdm09			TS4348 BD-like H3N2	
	Sero† (%)	GMT (95% CI)		Sero† (%)	GMT (95% CI)		Sero† (%)	GMT (95% CI)		Sero† (%)	GMT (95% CI)		Sero† (%)	GMT (95% CI)
<10	7/33 (21.2)	11 (7–16)		3/33 (9.1)	7 (5–9)		2/33 (6.1)	8 (6–10)		21/33 (63.6)	63 (34–119)		18/33 (54.5)	28 (15–51)
11–20	3/42 (7.1)	8 (6–9)		2/42 (4.8)	7 (5–9)		1/42 (2.4)	7 (6–8)		30/42 (71.4)	54 (36–81)		37/42 (88.1)	115 (81–162)
21–30	3/38 (7.8)	8 (6–10)		10/38 (26.3)	13 (8–19)		4/38 (10.5)	8 (6–10)		23/38 (60.5)	34 (22–52)		35/38 (92.1)	154 (106–225)
31–40	4/42 (9.5)	7 (6–9)		6/42 (14.3)	9 (7–12)		6/42 (14.3)	10 (8–14)		17/42 (40.5)	20 (13–29)		27/42 (64.3)	40 (27–59)
41–50	9/40 (22.5)	11 (8–15)		5/40 (12.5)	7 (5–10)		6/40 (15)	9 (7–13)		13/40 (32.5)	14 (10–21)		24/40 (60)	33 (23–48)
51–60	3/40 (7.5)	7 (5–10)		3/40 (7.5)	7 (5–9)		2/40 (5)	8 (6–11)		7/40 (17.5)	10 (7–15)		19/40 (47.5)	28 (18–42)
61–70	1/39 (2.5)	6 (5–7)		2/39 (5.1)	6 (5–8)		1/39 (2.6)	7 (6–8)		11/39 (28.2)	12 (8–17)		21/39 (53.8)	27 (19–38)
>70	4/79 (5.1)	7 (6–7)		8/79 (10.1)	8 (7–10)		5/79 (6.3)	7 (6–8)		24/79 (30.4)	15 (12–20)		58/79 (73.4)	54 (42–68)

*Serum samples were collected during 2013–2014 in Hong Kong and during 2015 in Guangzhou. BD, Binh Duong; EA, Eurasian–avian-like; GMT, geometric mean titer; sero, seroprevalence; TRIG, triple-reassortant internal gene. †Proportion of persons with hemagglutination inhibition antibody titers >1:40.

### Assessment of Population Immunity 

**Figure 2:**
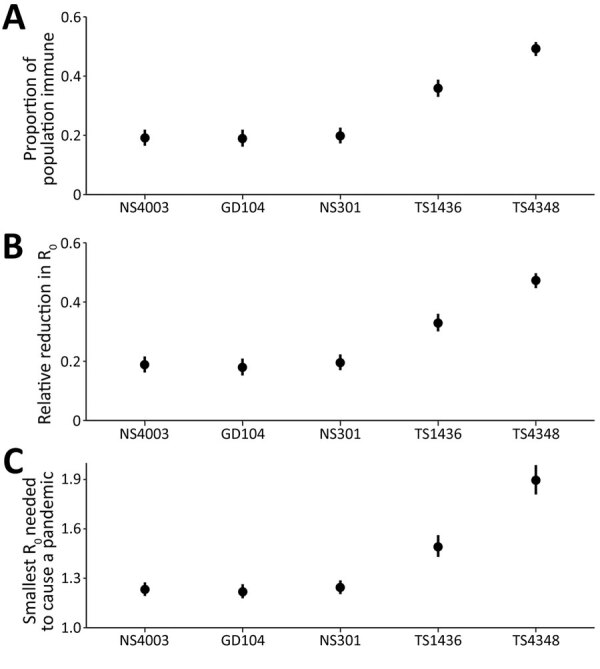
Estimations of overall population-level immunity against H1 and H3 viruses and the potential effect of population immunity on reproduction number in study to determine existing human population immunity as part of assessing influenza pandemic risk. Error bars represent the 95% credible intervals of the estimates. Data are shown from A/Swine/Hong Kong/NS4003/2016 (EA, H1N1) (NS4003), A/Swine/Guangdong/104/2013 (EA, H1N1) (GD104), A/Swine/Hong Kong/NS301/2013 (TR, H1N2) (NS301), A/Swine/Hong Kong/1436/2016 (pdmH1N1) (TS1436), and A/Swine/Hong Kong/4348/2016 (BD-like H3N2) (TS4348).

From our estimates of overall population immunity against different H1 and H3 swine influenza viruses and its potential effect on R_0_ and R_t_ (Figure 2), we determined that after weighting the protection conferred by each HAI titer level and by age distribution using the population age structure, only ≈19%–20% of the population was immune to A/swine/HK/NS4003/2016, A/swine/GD/104/2013, and A/swine/HK/NS301/2013 viruses (Appendix Table 2). We used a social contact matrix for Hong Kong to parametrize our estimates (Figure 2). We estimated that the population immunity in Guangzhou and Hong Kong would reduce R_0_ of A/swine/HK/NS4003/2016, rg-A/swine/GD/104/2013, or A/swine/HK/NS301/2013 by only ≈18%–20%. Because the smallest R_0_ needed to cause a pandemic is in the 1.22−1.24 range, if viruses with any of these HAs were to emerge in a form efficiently transmissible in humans, the cross-reactive human population immunity would impede its spread only modestly (Figure 2). 

In contrast, if A/swine/HK/4348/2016 (H3N2) were to acquire efficient biological transmissibility among humans, ≈49% of the population would be immune, which would suppress the inherent transmissibility of the virus by 47%; a pandemic would be prevented if the R_0_ of the emergent virus was <1.9 (95% CrI 1.81–1.99) (Figure 2). The H1N1pdm09-like A/swine/HK/1436/2016 (H1N1) virus would spread globally if R_0_ was >1.49 (95% CrI 1.43–1.56). In fact, antigenically drifted A/Michigan/45/2015-like viruses formed a subclade 6B.1A and continued to spread as seasonal H1N1 influenza during 2017–2020 (*31*). The estimates of reproduction numbers for seasonal influenza viruses are ≈1.28 (interquartile range 1.19–1.37) (*32*).

We have also presented the analysis of the data for the populations of Hong Kong and Guangzhou considered separately (Appendix Table 1); the results were very similar, and statistically significant differences were seen only with A/swine/HK/4348/2016 (H3N2). Guangzhou, compared with Hong Kong, showed significantly higher population immunity to A/swine/HK/4348/2016, providing a greater reduction in R_0_. 

For a sensitivity analysis, we investigated how critical the social contact matrix data were to the final outcome, by using the UK social contact matrix instead of the matrix for Hong Kong as a comparison model (*25*) (Appendix Table 2). The modeled estimates with the 2 contact matrixes gave similar results; we observed statistically significant differences only for A/swine/HK/1436/2016 (H1N1). Using the UK social contact matrix led to a significantly greater reduction in R_t_, attributable to higher-contact frequencies in child and young adult populations in the United Kingdom.

The H1N1pdm09 virus caused a pandemic in 2009 even though there were some cross-reactive HAI antibodies in older adults. Using serum samples collected before the spread of H1N1pdm09 in Hong Kong, we showed that only ≈12% (95% CrI 10%–14%) of the general population was immune to the pandemic virus (A/California/4/2009) before the first pandemic wave (Tables 3, 4). R_0_ would only have been reduced by ≈12% (95% CrI 10%–14%) and the smallest R_0_ needed for the virus to cause a pandemic was 1.13 (95% CrI 1.11–1.16), indicating the virus would spread readily in the population, as it did in 2009. Sensitivity analysis done with the UK contact matrix showed very similar results (Appendix Table 3). A previous study showed that >40% of children were infected in that first pandemic wave, confirming the low population immunity before exposure to this virus (*33*).

**Table 3:** Seroprevalence and geometric mean titers of hemagglutination inhibition antibodies to historical H2 and H1 pandemic viruses based on age group among persons in Hong Kong, China*

Age group, y	A/California/4/2009 (H1N1pdm09)†			A/Singapore/1/1957 (H2N2pdm1957)	
	Seroprevalence† (%), n = 600	GMT (95% CI)		Seroprevalence† (%), n = 295	GMT (95% CI)
0–10	0/72 (0)	6 (6–7)		0/24 (0)	5 (5–6)
11–20	10/107 (9.3)	8 (7–9)		0/38 (0)	5 (5–6)
21–30	3/46 (6.5)	6 (5–8)		0/39 (0)	5
31–40	5/39 (12.8)	8 (5–11)		0/37 (0)	5 (5–6)
41–50	9/125 (7.2)	6 (5–7)		13/38 (34.2)	15 (9–24)
51–60	6/131 (4.6)	6 (5–6)		40/40 (100)	243 (172–342)
61–70	1/54 (1.9)	6 (5–7)		40/40 (100)	320 (249–411)
>70	3/26 (11.5)	7 (5–10)		36/39 (92.3)	136 (89–209)

*GMT, geometric mean titer. †Proportion of persons with hemagglutination inhibition antibody titers >1:40.

**Table 4:** Estimations of overall population-level immunity against historical H2 and H1 pandemic viruses and the potential effect of population immunity on reproduction number among persons in Hong Kong, China*

Virus strain	Proportion of population immune (95% CI)	Relative reduction in R_0_ (95% CI)	Smallest R_0_ needed to cause pandemic (95% CI)
A/Singapore/1/1957 (H2N2)	0.37 (0.346–0.394)	0.321 (0.295–0.348)	1.472 (1.419–1.535)
A/California/04/2009 (H1N1)	0.117 (0.098–0.14)	0.115 (0.096–0.138)	1.13 (1.106–1.16)

*Serum samples for testing antibodies to the 1957 virus were collected in 2011 and those for testing antibodies to the 2009 virus were collected in 2008–2009.

From a previous study (*9*), we retrieved the HAI data for A/Singapore/1/1957 (H2N2) for 295 serum samples collected from children and adults in Hong Kong during August–December 2011 and reassessed population immunity using the methods from this study and the social contact matrices from Hong Kong (Tables 3, 4) and the United Kingdom (Appendix Table 3). Although ≈37% of the general population was immune to A/Singapore/1/1957 using either contact matrix, the resulting R_0_ was 1.47 when using the Hong Kong social matrix and 1.23 when using the UK social matrix. The highly skewed age-dependent population immunity profile was markedly more sensitive to the social contact patterns in the matrices.

## Discussion

We report a systematic approach for using a broad range of HAI titers in age-stratified serum samples together with data from social contact matrices to assess population immunity to viruses of pandemic concern. This approach is especially relevant in assessing risk from swine influenza viruses because levels of cross-reactive antibodies to the H1 and H3 virus subtypes vary in humans. A main reason why the H1N2 TRIG viruses, which provided the HA gene segment for the 2009 pandemic virus, were not regarded as pandemic candidates before the 2009 outbreak began, despite causing repeated previous zoonotic infections in North America, was the lack of consideration of the consequences of the low population immunity to this virus. 

The estimated median R_0_ was 1.8 for the 1918 pandemic, 1.65 for the 1957 pandemic, 1.8 for the 1968 pandemic, and 1.46 for the 2009 pandemic (*32*). We demonstrated that existing population immunity at the time of the emergence of the 2009 pandemic was low, which would enable the H1N1pdm09 virus to cause a pandemic if R_0_ was >1.13; estimated R_0_ was ≈1.46, and it did spread as a pandemic. EA H1N1 or TRIG H1N2 swine viruses now circulating in China (*11*,*13*) would face similarly low resistance from human population immunity if they were to become transmissible among humans. This finding is of particular concern because some of these viruses have 6 gene segments of H1N1pdm09 origin and are therefore potentially well adapted to human transmission (*13*). EA-lineage swine viruses have caused sporadic zoonotic infections in China, including one in which a case-patient died (*34*–*39*). One EA H1N1 virus in our study, A/Sw/HK/NS4003/2016, is of the predominant emergent EA reassortant genotype 4 (Appendix Figure 1), which was shown to have increased human infectivity (*40*). The HA1 amino acid sequences of A/Sw/HK/NS4003/2016 are similar to those of the representative genotype 4 virus A/swine/Shandong/1207/2016, with 97.9% aa identity and only 1 amino acid change (N74K, H1 numbering) in the Cb antigenic site. These 2 viruses thus pose substantial pandemic threats. In contrast, the swine Binh Duong-lineage H3N2 viruses, although they also have 6 H1N1pdm09 internal gene segments (*13*,*14*), would not cause a pandemic unless the virus had an R_0_ >1.9, a much less likely situation.

We found comparable age-stratified seroprevalence in Hong Kong and Guangzhou. In an earlier study, we reported similar seroprevalence to human and avian H2N2 viruses in the United States and Hong Kong (*9*). Studies in a few large cities worldwide might provide data relevant to other large urban population centers worldwide. Whereas differences in social contact matrixes (e.g., Hong Kong vs. the United Kingdom) may have had some influence on the overall conclusions, they might not dramatically change the conclusions about the pandemic risk of a virus, unless there was a skewed age distribution of antibody prevalence, such as with the H2N2 virus.

Among our study’s limitations was that we used HAI antibodies as our sole correlate of protection. Other protective mechanisms, including neuraminidase-inhibiting antibodies, HA stalk-binding antibodies, antibody-dependent cell cytotoxicity, and T-cell immune responses, would also provide measures of protection levels (*41*–*44*). However, quantitative measures of protection conferred by those immune correlates are lacking, precluding the use of similar approaches to assess their potential contributions to population immunity. Therefore, our estimates based on HAI alone provide a minimal assessment of population immunity to a given virus. Second, our estimates focused on emergence risk for a pandemic, not severity or effect. For example, because older adults were exposed to drift variants of H1N1 antigenically closer to the 1918 H1N1 pandemic virus, and because the 2009 H1N1 pandemic virus acquired the H1 from triple reassortant swine influenza viruses that had an HA closely related to the 1918 H1N1 virus, older adults had more cross-protective immunity against the H1N1pdm09 virus than did children and young adults, which reduced the overall infection rates as well as severe disease and death (*45*). Third, the serum samples used in this study were collected during 2013–2015; the population immunity profile may have changed since then.

However, our main aim in this report was to provide a quantitative approach for assessing population immunity, which is a key element in determining pandemic risk from influenza viruses. This approach identified several swine viruses that need full risk assessment. Some of these viruses have 5 or 6 internal gene segments derived from H1N1pdm09 viruses, which are well adapted to humans and have efficient binding to human receptors (as do most swine influenza viruses) and to which there is low human population immunity. Changes in hemagglutinin or neuraminidase or the balance between them (*46*) may be sufficient to make them efficiently transmissible between humans and therefore pandemic threats.

Appendix Additional information on assessing population immunity for determining risk for swine influenza pandemics.
