# Supplementary material for: Determining Existing Human Population Immunity as Part of Assessing Influenza Pandemic Risk
Source: Emerg Infect Dis. 2022 May;28(5):977–85. doi: 10.3201/eid2805.211965 (PMC9045452; doi:10.3201/eid2805.211965)
Supplement: Appendix — Additional information on assessing population immunity for determining risk for swine influenza pandemics. [file 21-1965-Techapp-s1.pdf]

Article DOI: https://doi.org/10.3201/eid2805.211965

Determining Existing Human Population Immunity as Part of Assessing Influenza Pandemic Risk

Appendix

Methods

Generation of Recombinant Virus

The hemagglutinin (HA) gene of wild-type GD104 virus (GenBank accession no. KJ725040) was synthesized using Invitrogen GeneArt Gene Synthesis (Thermo Fisher Scientific; https://www.thermofisher.com) and amplified by PCR with HA-gene–specific primers (*1*). The PCR product was cloned into the pHW2000 vector as described elsewhere (*2*,*3*). The genetic sequence of the HA plasmid was verified by Sanger sequencing.

The recombinant virus A/PR/8/34^{PB2,PB1,NP,NA,M,NS} x A/swine/Guangdong/104/2013^{HA} (Rg- PR8 x GD104^{HA}), which contains the HA gene derived from A/swine/Guangdong/104/2013 (H1N1) (GD104) and the 7 other genes from A/PR/8/34 (H1N1), was generated as described elsewhere (*3*). Briefly, 1 µg of each of the 8 segment plasmids (PB2, PB1, PA, HA, NP, NA, M, NS) were mixed with Opti-MEM medium and TransIT-LT1 Transfection Reagent (Mirus Bio LLC; https://www.mirusbio.com). The mixture was transfected into 293T cells prepared in 6-well plates. At day 3 after transfection, the transfection supernatant was added into MDCK cells and incubated for 72 hours. The tissue culture supernatants were harvested and hemagglutination testing was performed to confirm successful rescue of the virus. The virus stocks were generated by passaging the tissue culture supernatants containing the recombinant virus GD104 into MDCK cells twice and the HA sequence of the stock virus GD104 was further confirmed by reverse transcription PCR and Sanger sequencing.

Reproduction Number Modeling

We partitioned the seroprevalence data into n = 8 age groups (0–10, 11–20, 21–30, 31–40, 41–50, 51–60, 61–70, >70 years) and m = 9 hemagglutination inhibition (HAI) titer levels

(<1:10, 1:10, 1:20, 1:40, 1:80, 1:160, 1:320, 1:640, ≥1:1280). We obtained the age distribution of population ρ_i from the most recent census data from Hong Kong (2016) and Guangzhou (2015). We first compared the age stratified seroprevalence in Hong Kong and Guangzhou to ascertain whether there were significant differences. We used data reported in an earlier study (*4*,*5*) from an experimental challenge of human volunteers in which they assessed the proportion of persons protected from infection at each HAI antibody titer to estimate the protection conferred by the serologic results observed in each person in our study to each of the viruses tested. To estimate the proportion of population in each HAI titer group for each age group, we used Bayesian inference with Dirichlet conjugates for multinomial likelihood $\frac{y_i}{x_{i1}!\dots x_{im}!}\prod_{j=1}^{m} s_{ij}^{x_{ij}}$, where s_{ij} was the proportion of age group i with the jth HAI titer, y_i was the number of persons in age group i in our serosurveys and x_{ij} was the number of subjects in age group i with the jth HAI titer (*6*). We assumed noninformative priors with parameters $\alpha_j = 1$ for all HAI titer level j, and hence the joint posterior distributions of $(s_{i1}, \dots, s_{im})$ were Dirichlet distributions with parameters $\alpha_{ij} = x_{ij} + 1$ for $j = 1, \dots, m$. As such, the proportion of the population that was immune could be obtained from $\sum_{i=1}^{n} p_{ij} \sum_{j=1}^{m} s_{ij} z_j$, where z_j was the seroprotection level from influenza conferred by the jth HAI titer level. We constructed the next-generation matrix $\{Q_{ij}\}$, where Q_{ij} was the average number of cases in age group i generated by a primary infection in age group j over the course of its infectious period, using the social contact matrix for Hong Kong from a previous study (*7*). Social contact matrix for the United Kingdom population (*8*) was also used as a sensitivity analysis to assess the effect of different contact matrixes on overall population immunity. Since the matrix $\{Q_{ij}\}$ was the contact frequency matrix sampled from a fully susceptible population, the basic reproduction number R_0 was defined as the largest eigenvalue of $\{Q_{ij}\}$ (*9*,*10*). Because the susceptible proportion of age group i was $1 - \sum_{j=1}^{m} s_{ij} z_j$, we then constructed another matrix $\{(1 - \sum_{j=1}^{m} s_{ij} z_j) Q_{ij}\}$, which only included susceptible population and thus the effective reproduction number R_e was the largest eigenvalue of this matrix. Given that population immunity profile, we calculated the corresponding relative reduction in transmissibility as $1 - \frac{R_e}{R_0}$. Since a pandemic could only continue to spread if its reproduction number is >1, we then computed the smallest R_0 needed to cause a pandemic for each test viruses as $1/1 - relative\ reduction\ in\ R_0$. The credible intervals for the parameters estimated were generated using 10,000 samples randomly drawn from the joint posterior distribution of

$(s_{i1}, \ldots, s_{im})$ for each age group i and the 95% credible intervals were the 2.5 and 97.5 percentiles of the 10,000 repeated estimates.

References

1. Hoffmann E, Stech J, Guan Y, Webster RG, Perez DR. Universal primer set for the full-length amplification of all influenza A viruses. Arch Virol. 2001;146:2275–89. PubMed https://doi.org/10.1007/s007050170002

2. Hoffmann E, Neumann G, Hobom G, Webster RG, Kawaoka Y. "Ambisense" approach for the generation of influenza A virus: vRNA and mRNA synthesis from one template. Virology. 2000;267:310–7. PubMed https://doi.org/10.1006/viro.1999.0140

3. Hoffmann E, Neumann G, Kawaoka Y, Hobom G, Webster RG. A DNA transfection system for generation of influenza A virus from eight plasmids. Proc Natl Acad Sci U S A. 2000;97:6108–13. PubMed https://doi.org/10.1073/pnas.100133697

4. Hobson D, Curry RL, Beare AS, Ward-Gardner A. The role of serum haemagglutination-inhibiting antibody in protection against challenge infection with influenza A2 and B viruses. J Hyg (Lond). 1972;70:767–77. PubMed

5. Nauta JJP, Beyer WEP, Osterhaus ADME. On the relationship between mean antibody level, seroprotection and clinical protection from influenza. Biologicals. 2009;37:216–21. PubMed https://doi.org/10.1016/j.biologicals.2009.02.002

6. Lunn D, Jackson C, Best N, Thomas A, Spiegelhalter D. The BUGS book: a practical introduction to Bayesian analysis. Boca Raton, FL: CRC Press, Taylor & Francis Group; 2013.

7. Leung K, Jit M, Lau EHY, Wu JT. Social contact patterns relevant to the spread of respiratory infectious diseases in Hong Kong. Sci Rep. 2017;7:7974. PubMed https://doi.org/10.1038/s41598-017-08241-1

8. Mossong J, Hens N, Jit M, Beutels P, Auranen K, Mikolajczyk R, et al. Social contacts and mixing patterns relevant to the spread of infectious diseases. PLoS Med. 2008;5:e74. PubMed https://doi.org/10.1371/journal.pmed.0050074

9. Diekmann O, Heesterbeek JA, Metz JA. On the definition and the computation of the basic reproduction ratio R0 in models for infectious diseases in heterogeneous populations. J Math Biol. 1990;28:365–82. PubMed https://doi.org/10.1007/BF00178324

10. Diekmann O, Heesterbeek JAP, Roberts MG. The construction of next-generation matrices for compartmental epidemic models. J R Soc Interface. 2010;7:873–85. PubMed https://doi.org/10.1098/rsif.2009.0386

11. Nguyen LT, Schmidt HA, von Haeseler A, Minh BQ. IQ-TREE: a fast and effective stochastic algorithm for estimating maximum-likelihood phylogenies. Mol Biol Evol. 2015;32:268–74. PubMed https://doi.org/10.1093/molbev/msu300

Appendix Table 1. Estimates of population-level immunity against different lineages of swine influenza viruses and the potential effect of population immunity on reproduction number based on Hong Kong and Guangzhou populations separately*

Swine influenza virus strain	Proportion of population immune (95% cri)		Relative reduction in reproduction no. (95% cri)		Smallest R_0 needed to cause a pandemic (95% cri)	
	Hong Kong†	Guangzhou‡	Hong Kong†	Guangzhou‡	Hong Kong†	Guangzhou‡
A/Swine/HK/NS4003/2016 (EA, H1N1)	0.227 (0.19–0.267)	0.256 (0.219–0.297)	0.228 (0.191–0.269)	0.248 (0.211–0.287)	1.295 (1.235–1.368)	1.329 (1.268–1.403)
rg-A/Swine/GD/104/2013 (EA, H1N1)	0.234 (0.196–0.274)	0.242 (0.202–0.284)	0.231 (0.193–0.273)	0.23 (0.192–0.272)	1.301 (1.239–1.376)	1.298 (1.237–1.374)
A/Swine/HK/NS301/2013 (TRIG, H1N2)	0.223 (0.187–0.262)	0.275 (0.238–0.314)	0.223 (0.186–0.264)	0.265 (0.23–0.302)	1.287 (1.228–1.359)	1.361 (1.299–1.433)
A/Swine/HK/1436/2016 (pdm2009, H1N1)	0.345 (0.308–0.383)	0.419 (0.382–0.455)	0.339 (0.3–0.379)	0.378 (0.341–0.416)	1.513 (1.428–1.611)	1.607 (1.517–1.711)
A/Swine/HK/4348/2016 (BD-like H3N2)	0.438 (0.404–0.471)	0.553 (0.522–0.581)	0.428 (0.392–0.463)	0.533 (0.503–0.561)	1.747 (1.644–1.863)	2.142 (2.014–2.276)

*CrI, credible intervals; BD, Binh Duong; EA, Eurasian–avian-like; TRIG, triple-reassortant internal gene
†Serum samples were collected in 2013–2014 in Hong Kong.
‡Serum samples were collected in 2015 in Guangzhou

Appendix Table 2. Estimates* of population-level immunity against different lineages of swine influenza viruses and the potential effect of population immunity on reproduction number based on the United Kingdom social contact matrix in the combined Hong Kong† and Guangzhou‡ study groups

Swine influenza virus strain	Proportion of population immune (95% CrI)	Relative reduction in reproduction no. (95% CrI)	Smallest R_0 needed to cause a pandemic (95% CrI)
A/Swine/HK/NS4003/2016 (EA, H1N1)	0.191 (0.166–0.22)	0.198 (0.166–0.232)	1.246 (1.2–1.302)
rg-A/Swine/GD/104/2013 (EA, H1N1)	0.189 (0.161–0.218)	0.18 (0.148–0.216)	1.219 (1.174–1.276)
A/Swine/HK/NS301/2013 (TRIG, H1N2)	0.198 (0.173–0.226)	0.195 (0.164–0.227)	1.242 (1.196–1.294)
A/Swine/HK/1436/2016 (pdm2009, H1N1)	0.359 (0.33–0.387)	0.41 (0.379–0.439)	1.696 (1.609–1.784)
A/Swine/HK/4348/2016 (BD-like H3N2)	0.492 (0.467–0.515)	0.51 (0.482–0.537)	2.042 (1.931–2.158)

*CrI, credible intervals**; BD,** Binh Duong; EA, Eurasian–avian-like; TRIG, triple-reassortant internal gene
†Serum samples were collected in 2013–2014 in Hong Kong.
‡Serum samples were collected in 2015 in Guangzhou.

Appendix Table 3. Estimates* of overall population-level immunity against historical H2‡ and H1† pandemic viruses and the potential effect of population immunity on reproduction number based on the United Kingdom social contact matrix

Swine influenza virus strain	Proportion of population immune (95% CrI)	Relative reduction in reproduction no. (95% CrI)	Smallest R_0 needed to cause a pandemic (95% CrI)
A/California/04/2009 (H1N1)†	0.118 (0.098–0.140)	0.127 (0.106–0.149)	1.145 (1.119–1.175)
A/Singapore/1/1957 (H2N2)‡	0.370 (0.346–0.395)	0.187 (0.156–0.226)	1.231 (1.185–1.292)

*CrI, credible intervals
†Serum samples for testing for H1N1pdm09 antibodies were collected in 2008–2009.
‡Serum samples for testing for H2N2pdm1957 antibodies were collected in 2011.

Swine influenza virus	PB2	PB1	PA	HA	NP	NA	M	NS
A/Swine/HK/NS4003/2016 (EA, H1N1)	pdm09	pdm09	pdm09	EA	pdm09	EA	pdm09	TRIG
rg-A/Swine/GD/104/2013 (EA, H1N1)	PR8	PR8	PR8	EA	PR8	PR8	PR8	PR8
A/Swine/HK/NS301/2013 (TRIG, H1N2)	pdm09	pdm09	pdm09	TRIG	pdm09	TRIG	pdm09	pdm09
A/Swine/HK/4348/2016 (BD-like H3N2)	pdm09	pdm09	pdm09	BD-like human	pdm09	BD-like human	pdm09	TRIG

Appendix Figure 1. Lineage origin of the 8 gene segments of the viruses studied. Genetic characteristics of the viruses used in this study.

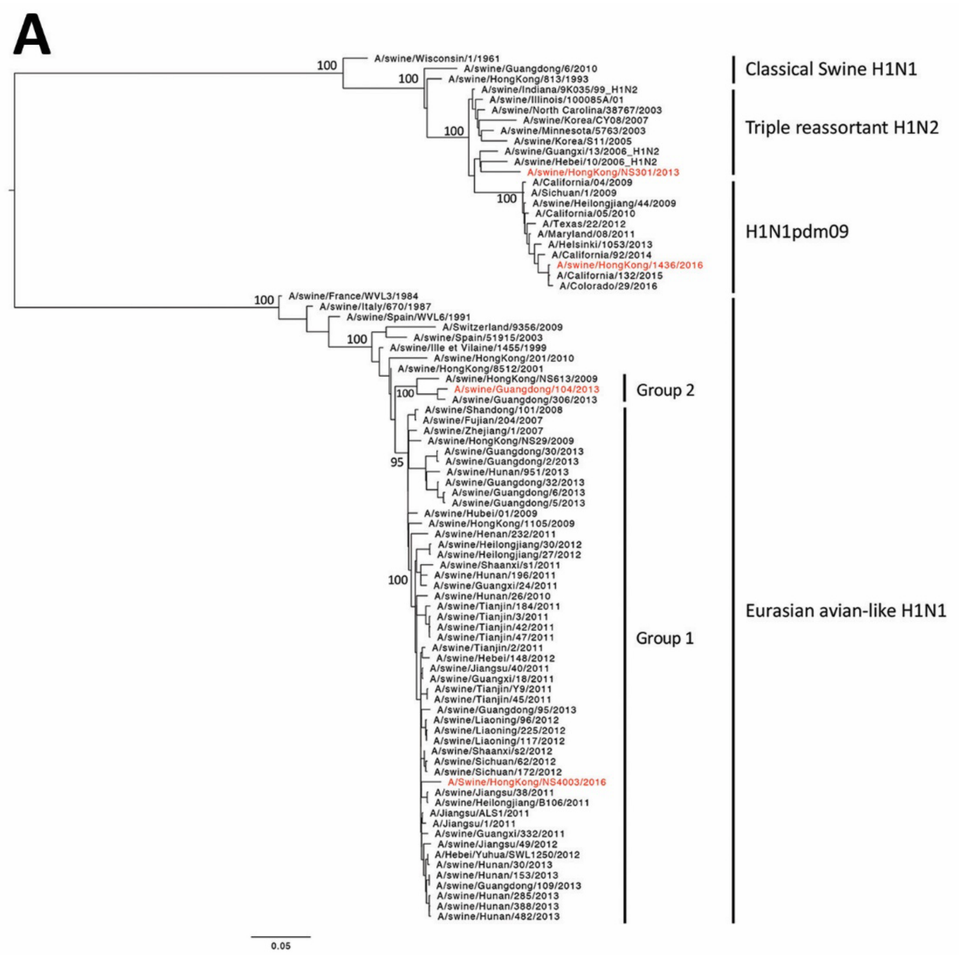

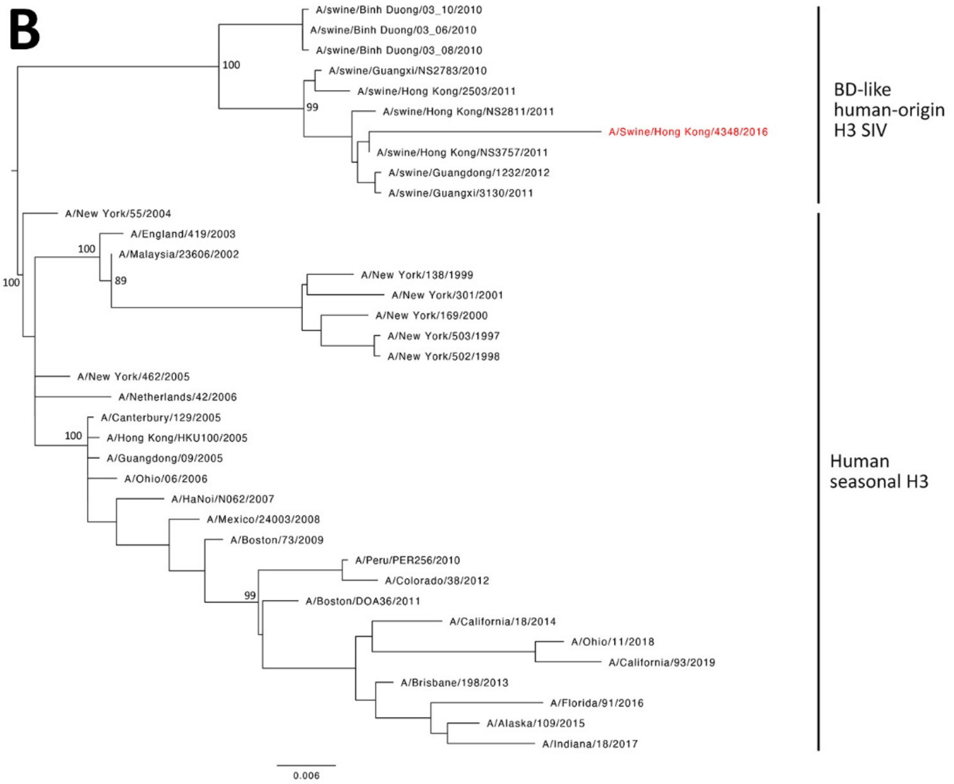

Appendix Figure 2. Phylogenetic relationships of the viruses used in this study. Phylogenetic tree of the viruses selected for hemagglutination inhibition assay. Maximum likelihood trees were constructed using IQ-TREE (*11*) based on the coding sequences at 33–1733 nt of the virus hemagglutinin (HA) and obtained branch supports with SH-aLRT test. A) HA genes for H1 swine influenza viruses. B) Phylogenetic tree of HA genes for H3 swine influenza viruses. Sequences of viruses with names in black were downloaded from available databases; viruses with names in red were selected as the virus antigens used for HAI assay. Scale bar indicates the number of nucleotide substitutions per site.
